# Who Does Well in Life? Conscientious Adults Excel in Both Objective and Subjective Success

**DOI:** 10.3389/fpsyg.2012.00356

**Published:** 2012-09-28

**Authors:** Angela L. Duckworth, David Weir, Eli Tsukayama, David Kwok

**Affiliations:** ^1^Department of Psychology, University of PennsylvaniaPhiladelphia, PA, USA; ^2^Institute for Social Research, University of MichiganAnn Arbor, MI, USA

**Keywords:** conscientiousness, personality, Big Five, income, wealth, subjective well-being

## Abstract

This article investigates how personality and cognitive ability relate to measures of objective success (income and wealth) and subjective success (life satisfaction, positive affect, and lack of negative affect) in a representative sample of 9,646 American adults. In cross-sectional analyses controlling for demographic covariates, cognitive ability, and other Big Five traits, conscientiousness demonstrated beneficial associations of small-to-medium magnitude with all success outcomes. In contrast, other traits demonstrated stronger, but less consistently beneficial, relations with outcomes in the same models. For instance, emotional stability demonstrated medium-to-large associations with life satisfaction and affect but a weak association with income and no association with wealth. Likewise, extraversion demonstrated medium-to-large associations with positive affect and life satisfaction but small-to-medium associations with wealth and (lack of) negative affect and no association with income. Cognitive ability showed small-to-medium associations with income and wealth but no association with any aspect of subjective success. More agreeable adults were worse off in terms of objective success and life satisfaction, demonstrating small-to-medium inverse associations with those outcomes, but they did not differ from less agreeable adults in positive or negative affect. Likewise, openness to experience demonstrated small-to-medium inverse associations with every success outcome except positive affect, in which more open adults were slightly higher. Notably, in each of the five models predicting objective and subjective success outcomes, individual differences other than conscientiousness explained more variance than did conscientiousness. Thus, the benefits of conscientiousness may be remarkable more for their ubiquity than for their magnitude.

## Introduction

Success in life can be defined either objectively or subjectively. Objective success entails doing well according to some common metric uniformly applied to all individuals in a society, whereas subjective success concerns an individual’s personal assessment of his or her life situation. Which matters more: how we stack up to others according to widely held standards or, rather, how we think and feel about our own lives? Persuasive arguments can be made on either side, suggesting that objective and subjective aspects of well-being are more usefully considered complementary than competing life outcomes (Forgeard et al., 2011). One possibility, unexamined by prior research, is that certain traits are of ubiquitous utility in the sense that they contribute to both objective *and* subjective success, whereas others entail tradeoffs in either domain. In the current investigation, we examine how Big Five personality traits and cognitive ability relate to lifetime earnings, wealth, life satisfaction, and emotional experience in a representative sample of American adults.

In a meritocratic society, earnings and wealth are arguably the clearest criteria by which to judge objective success. While not the only objective outcome, nor even the most important, money is a monotonically positive good: all other things being equal, most people would prefer to have more money than less. Accordingly, individuals in the labor market compete for higher (not lower) wages and wealth. In terms of subjective success, three dimensions of well-being are distinguishable: life satisfaction, positive affect, and (the absence of) negative affect (Pavot and Diener, 2011). Positive affect includes states such as cheerful and satisfied, and negative affect includes states such as hopeless and nervous. Life satisfaction refers to the overall reflective, evaluative appraisal of one’s life, exemplified by the commonly asked survey question, “How satisfied are you with your life as a whole these days?” Global assessments of one’s quality of life, according to whatever standards individuals set for themselves, elicit evaluation not just of one narrow domain (e.g., job satisfaction, marital satisfaction) but rather all facets weighed idiosyncratically and considered by an individual in totum. These three aspects of subjective well-being are only moderately correlated and belong to overlapping but distinct nomological nets (Diener, 1984; Pavot and Diener, 2008). For instance, life satisfaction demonstrates slightly stronger associations with income than does either positive or negative affect (Kahneman and Deaton, 2010; Sacks et al., 2010).

Historically, cognitive ability has drawn attention from psychologists interested in universally beneficial traits (Gottfredson, 2002). In addition to superior performance in school (Neisser et al., 1996), more intelligent individuals have been shown to earn higher incomes (Heckman et al., 2006). But are the more able-minded also better off in terms of how they think and feel about their own lives? Hemingway’s observation that (Hemingway, 1986), “Happiness in intelligent people is the rarest thing I know” (p. 86) suggests an inverse relation between cognitive ability and happiness, but empirical findings on this topic are mixed (Sigelman, 1981; Judge et al., 2010). Probably the most uncontroversial conclusion at this point is that cognitive ability predicts objective success more strongly and reliably than it predicts subjective success.

Less is known about how personality traits contribute to objective success (Farkas, 2003). Ironically, the importance of personality to achievement, particularly beyond the four walls of the classroom, was abundantly clear to pioneers in intelligence testing (Binet and Simon, 1916; Wechsler, 1940). Unfortunately, early empirical efforts in this direction (e.g., Jencks, 1979) did not add up to much, primarily because personality psychology lacked a taxonomy that would “permit researchers to study specified domains of personality characteristics, rather than examining separately the thousands of particular attributes that make each human being individual and unique”(John and Srivastava, 1999, p. 102). In recent decades, most personality psychologists have come to agree that a five-factor model encompassing conscientiousness, agreeableness, extraversion, emotional stability, and openness to experience, organizes personality traits at the broadest level of abstraction (John et al., 2008). More systematic research made possible by this framework suggests that relative to other Big Five traits, conscientiousness is the most reliable predictor of academic course grades (Poropat, 2009), physical health (Roberts et al., 2005; Deary et al., 2010; Kern and Friedman, 2011), longevity (Deary et al., 2008; Kern and Friedman, 2008), job performance (Barrick et al., 2001), and marital stability (Roberts et al., 2007).

A recent meta-analysis of 19 studies associating Big Five personality traits to earnings found a positive association between conscientiousness and earnings, a relationship that was significantly stronger in longitudinal studies (corrected *r* = 0.16) than in cross-sectional studies (corrected *r* = 0.03; Roberts et al., 2011). Likewise, a meta-analysis of associations between Big Five personality dimensions and subjective well-being established that conscientiousness is positively associated with life satisfaction (corrected *r* = 0.27) and positive affect (corrected *r* = 0.31) and inversely associated with negative affect, corrected *r* = −0.26 (Steel et al., 2008). Notably, Big Five extraversion and emotional stability also demonstrated small-sized associations with earnings (corrected *r* = 0.11 and 0.14, respectively) and stronger associations with aspects of subjective well-being (absolute values for corrected *r*s from 0.23 to 0.64) in these meta-analytic investigations.

The current investigation extends prior research on personality and success in several ways. First, income in prior studies has typically been self-reported, leaving open the possibility that observed associations are inflated by common method bias. For instance, of the 19 studies included in the Roberts et al. (2011) meta-analysis, 15 used self-reported data for income. Second, whereas numerous studies have related individual differences to either objective *or* subjective success, almost none have investigated both types of success in a common sample. Thus, inferences about how traits may relate differentially to either type of success outcome are limited. Similarly, studies of personality tend not to include measures of cognitive ability and vice versa (Hofstee, 2001), precluding a direct comparison of the predictive validities of personality vs. cognitive ability. Moreover, since openness to experience is moderately associated with cognitive ability (McCrae and Costa, 1997), many studies cannot rule out cognitive ability as a third-variable confound for relationships between openness and outcomes. Finally, to our knowledge, no prior study relating personality to either objective or subjective success has used a large, national sample of American adults.

In this study, we examine how Big Five personality traits and cognitive ability relate to aspects of objective and subjective success in a national sample of American adults. Our measures include self-report personality and subjective well-being questionnaires, four tests of cognitive ability, income data from Social Security records, and data on wealth from structured interviews. In separate models predicting earned income, household wealth, life satisfaction, positive affect, and negative affect, we control for demographic covariates including gender, ethnicity, age, and years of education. Furthermore, in all models, we correct for measurement error by using latent factors for psychological constructs (i.e., personality traits, cognitive ability, and aspects of subjective well-being).

## Materials and Methods

### Participants

Participants were drawn from the Health and Retirement Study (HRS), a national probability sample of American adults over age 50 and their spouses initiated in 1992 but refreshed every 6 years with additional cohorts of participants. About three-fourths of HRS participants signed consents for access to their Social Security earnings histories, a subset which is generally representative of the complete sample (Haider and Solon, 2000). Using listwise deletion on the predictor, income, and wealth variables yielded a final sample of *N* = 9,646 (mean age = 68 years, range = 30–91). About 83% of participants were White, 11% were African American, and 6% Hispanic; 58% were female. A subset of *N* = 5,000 married participants were used for wealth analyses.

### Procedure

In 2006, a collective 82% of HRS participants participated in a face-to-face interview and received a self-report questionnaire, which they were asked to complete and mail to the University of Michigan. The overall response rate was 74%.

### Measures

#### Personality

Personality was assessed with the Midlife Development Inventory personality scales (Lachman and Bertrand, 2001). Participants used a 4-point rating scale (1 = *not at all*, 4 = *a lot*) to indicate how well each of 26 trait adjectives described themselves. For example, Big Five conscientiousness was assessed with the items “organized,” “responsible,” “hardworking,” “careless” (reverse-scored), and “thorough.” Coefficient alphas for Big Five subscales ranged from 0.67 for conscientiousness to 0.82 for agreeableness.

#### Cognitive ability

Cognitive ability was assessed with a multidimensional measure based on the Telephone Interview for Cognitive Status (Brandt et al., 1988) and included tests of episodic memory (sum of immediate and delayed word recall), working memory (in a backward counting task), numeracy, and vocabulary. When repeated measures of these variables were available, we used participants’ earliest observation to minimize the impact of age-related decline. For a validation study of the HRS cognitive ability battery, see Herzog and Wallace (1997).

#### Objective success

Lifetime income (*M* = $980,000, SD = $738,000) was calculated using the average indexed monthly earnings in Social Security-linked records and adjusted to constant dollars of 2006 using the same wage index. Because annual Social Security earnings are capped at a taxable maximum, the observed distribution was not sufficiently skewed to require log-transformation to approximate normality. Wealth (*M* = $725,000, SD = $2,463,000) was measured in structured interviews by an extensive series of detailed questions about various types of household assets. For a review of the design considerations and validity of the HRS wealth estimates, see Smith (1995). To reduce skew, we natural log-transformed household wealth for all analyses (*M* = 12.52, SD = 1.52).

#### Subjective success

Subjective success was evaluated using separate self-report questionnaires for positive affect, negative affect, and life satisfaction. Positive and negative affect were measured using scales developed for the Midlife Development Inventory (Mroczek and Kolarz, 1998). Specifically, participants used a 5-point rating scale (1 = *all of the time*, 5 = *none of the time*) to indicate how frequently in the last month they had experienced six positive mood states (e.g., “cheerful”) and six negative mood states (e.g., “so depressed that nothing could cheer you up”). Life satisfaction was measured using the Satisfaction with Life Scale (Diener et al., 1985). Participants endorsed five items (e.g., “in most ways, my life is close to ideal”) using a 6-point scale (1 = s*trongly disagree*, 6 = s*trongly agree*). Coefficient alphas for positive affect, negative affect, and life satisfaction were 0.92, 0.88, and 0.90, respectively.

### Analytic strategy

We used structural equation modeling (SEM) in Mplus to correct for measurement error, using latent factors for psychological variables (i.e., Big Five personality traits, cognitive ability, positive affect, negative affect, and life satisfaction). However, we treated income and wealth as observed variables in their respective models because only one indicator was available for each.

**Figure 1:**
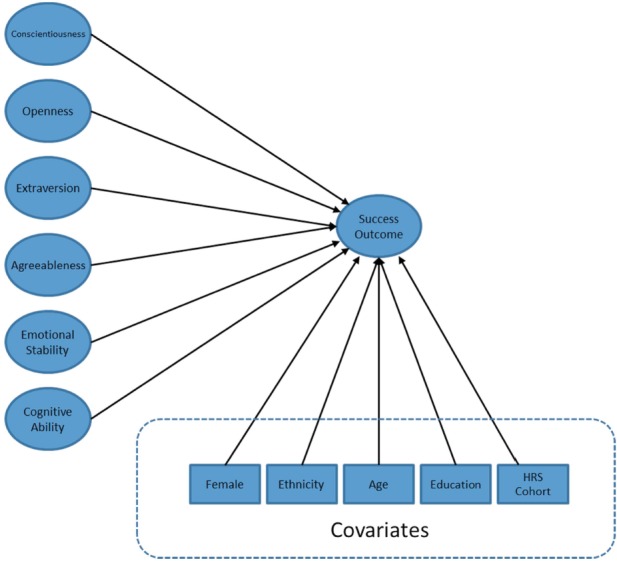
**Schematic of structural equation models**. Latent variable indicators, covariances among predictors, error variances, dummy variables, and disturbances are not displayed. Success outcomes include income and wealth (observed variables), and positive affect, negative affect, and life satisfaction (latent variables).

We first examined bivariate associations among personality traits, cognitive ability, and success outcomes. Next, because many of these associations were substantial in magnitude, we estimated the variance uniquely explained by each trait by fitting separate SEM models predicting positive affect, negative affect, life satisfaction, income, and wealth (see Figure 1). To reduce the possibility of third-variable confounds, we included the demographic variables of gender, ethnicity, age, years of education, and HRS study cohort as covariates in these analyses. Each model had all predictors included simultaneously, and all predictors were allowed to correlate. The wealth model had couples as the level of analysis with couples’ wealth as the outcome, husband and wife personality and cognitive ability as predictors, and husband and wife income as an additional covariate. While separate husband and wife variables were included as predictors, a model constraining their effects to be equal fit as well as did an unconstrained model, χ^2^(6) = 2.63, *p* = 0.85. Therefore, we report a single set of results for husbands and wives from the constrained model. Summary statistics and bivariate correlations for all observed variables are available upon request.

## Results

Descriptive statistics and bivariate associations for trait and success outcome variables are displayed in Table 1. Cognitive ability predicted income (*r* = 0.34) and wealth (*r* = 0.40) better than did any personality trait (absolute *r*s from 0.04 to 0.20). In contrast, personality traits typically predicted all three dimensions of subjective well-being better than did cognitive ability. For instance, positive affect was associated with each of the Big Five personality traits (*r*s from 0.46 to 0.63), but its association with cognitive ability was smaller (*r* = 0.21). As expected, negative affect was inversely correlated with emotional stability (*r* = −0.75) but also inversely correlated with other Big Five traits (*r*s from −0.13 to −0.29) more so than with cognitive ability (*r* = −0.12). Finally, life satisfaction was associated more strongly with all Big Five traits (*r* = 0.17–0.36) than with cognitive ability (*r* = 0.16).

**Table 1:** **Means, standard deviations, and correlations for personality, cognitive ability, and success variables**.

	*M*	SD	1	2	3	4	5	6
Conscientiousness	2.56	0.48	–					
Openness	1.95	0.55	0.67***	–				
Extraversion	2.20	0.55	0.61***	0.68***	–			
Agreeableness	2.53	0.47	0.63***	0.51***	0.80***	–		
Emotional stability	2.71	0.61	0.23***	0.20***	0.25***	0.07***	–	
Cognitive ability	0.00	1.00	0.27***	0.27***	0.01	0.02	0.16***	–
Income	$980,000	$738,000	0.08***	0.10***	−0.04**	−0.14***	0.16***	0.34***
Wealth	$725,432	$2,463,367	0.20***	0.14***	0.07***	0.02	0.15***	0.40***
Positive affect	4.26	0.62	0.56***	0.56***	0.63***	0.48***	0.46***	0.21***
Negative affect	2.41	0.79	−0.29***	−0.17***	−0.28***	−0.13***	−0.75***	−0.12***
Life satisfaction	4.84	1.59	0.29***	0.21***	0.31***	0.17***	0.36***	0.16***

*Means and standard deviations for personality and subjective well-being variables are based on unit-weighted subscales, whereas correlations are based on latent variables. The mean and standard deviation for wealth are from the untransformed variable, whereas correlations involving wealth are from a model using log-transformed wealth correlated with husband and wife variables whose effects were constrained to be equal (e.g., the correlation between husband conscientiousness and wealth and the correlation between wife conscientiousness and wealth were constrained to be equal). *N* = 9,646. Correlations involving wealth had a subset of *n* = 5,000 married participants*.

**p < 0.01, ***p < 0.001.

Consistent with prior research, personality traits were substantially intercorrelated (average *r* = 0.50). In general, associations between cognitive ability and personality traits were weaker (average *r* = 0.15), but in some cases medium in size (e.g., correlations with openness to experience and conscientiousness were both *r*s = 0.27). Thus, to clarify relationships between trait and success outcomes, we fit simultaneous SEM models in which all traits were entered as predictors at once, along with demographic covariates. These simultaneous models yielded estimates for the variance in success outcomes uniquely explained by each trait, when controlling for demographics. Fit statistics for all five models were acceptable: RMSEAs and SRMRs ≤ 0.06; CFIs ≥ 0.81^1^. Absolute factor loadings ranged from 0.27 to 0.89 (average = 0.61), *p*s < 0.001.

As shown in Table 2, in simultaneous models controlling for cognitive ability, demographics, and other personality traits, conscientious adults earned more money (β = 0.13) over their lifetimes and, when controlling for lifetime income, ended up with more savings (β = 0.16). While these effect sizes were small in magnitude, they were nevertheless substantively consequential. To illustrate, adults who were a standard deviation higher than average in conscientiousness earned an additional $96,000 over their lifetimes^2^. Controlling for earnings, husbands and wives who were each a standard deviation higher in conscientiousness accumulated $171,000 more in savings than the average American household^3^.

**Table 2:** **Standardized regression coefficients and standard errors from separate structural equation models for each outcome of objective and subjective success**.

	Objective success				Subjective success					
	Lifetime income		wealth^a^		Positive affect		Negative affect		Life satisfaction	
	β	SE_β_	β	SE_β_	β	SE_β_	β	SE_β_	β	SE_β_
Conscientiousness	0.13***	0.02	0.16***	0.03	0.17***	0.03	−0.20***	0.03	0.20***	0.03
Openness	−0.07**	0.02	−0.08**	0.03	0.10**	0.03	0.18***	0.03	−0.17***	0.04
Extraversion	0.00	0.03	0.16**	0.05	0.38***	0.04	−0.15***	0.04	0.41***	0.05
Agreeableness	−0.06*	0.03	−0.18***	0.04	−0.01	0.04	0.06	0.04	−0.21***	0.05
Emotional stability	0.04**	0.01	0.00	0.02	0.31***	0.02	−0.70***	0.01	0.26***	0.02
Cognitive ability	0.21***	0.02	0.14***	0.02	0.03	0.03	0.02	0.03	0.05	0.03
Personality Δ*R*^2^	0.01		0.04		0.48		0.55		0.19	
Cognitive ability Δ*R*^2^	0.02		0.02		0.00		0.00		0.00	
*R*^2^	0.39		0.34		0.56		0.60		0.24	

*Standardized regression coefficients are from separate models for each outcome with predictors included simultaneously. *N* = 9,646 for all models except for Wealth, which had a subset of *n* = 5,000 married participants*.

*^a^In this model, couples’ wealth was predicted from husband and wife variables simultaneously; effects were found to be equivalent*.

*p < 0.05, **p < 0.01, ***p < 0.00.

Likewise, adults who were higher in cognitive ability earned more money (β = 0.21) and, when controlling for lifetime income, ended up with more savings (β = 0.14). However, for other personality traits, associations with earnings and wealth were less uniformly positive. For instance, emotional stability demonstrated a weak association with income but no association with wealth. Likewise, extraversion demonstrated a small-to-medium association with wealth but no association with income. Notably, openness to experience, which demonstrated small but positive bivariate associations with income and wealth, was in simultaneous models including cognitive ability and demographic covariates slightly inversely correlated with the same measures of objective success. Likewise, in simultaneous models, agreeableness demonstrated small-to-medium inverse associations with income and wealth.

Conscientiousness showed small-to-medium associations with all dimensions of subjective success. Specifically, adults who were a standard deviation higher in conscientiousness than demographically similar peers were about 0.20 standard deviations higher in positive affect, (lack of) negative affect, and life satisfaction. As expected, extraversion demonstrated medium-to-large associations with both positive affect and life satisfaction and a small (inverse) association with negative affect. Similarly, emotional stability showed medium associations with positive affect and life satisfaction and a large association with negative affect. Agreeableness was unrelated to either positive or negative affect but, surprisingly, demonstrated a small-to-medium inverse association with life satisfaction. Finally, openness to experience demonstrated a small positive association with positive affect but also a small-to-medium association with negative affect and an inverse association with life satisfaction. Contrariwise, cognitive ability did not explain significant variance in any dimension of subjective success.

## Discussion

In a national sample of 9,646 American adults, Big Five conscientiousness demonstrated consistently beneficial associations with both objective and subjective success. More conscientious adults earned and saved more money, even when controlling for other Big Five traits, several important demographic covariates and cognitive ability. Likewise, more conscientious adults were more satisfied with their lives and experienced more positive and less negative emotion. While other traits explained more variance in particular outcomes, their benefits were less consistent across success outcomes. For instance, cognitive ability was a stronger predictor of income and comparably predictive of wealth but was unrelated to life satisfaction and positive and negative affect. Extraversion was a better predictor of positive affect and life satisfaction and comparably predictive of wealth but was unrelated to income. Emotional stability was more predictive of all aspects of subjective success but was only slightly predictive of income and unrelated to wealth.

Consistent with the adage about nice guys finishing last, more agreeable adults actually earned and saved less money and were less satisfied with their lives. The observed negative association with life satisfaction may seem surprising given Steel et al.’s (2008) meta-analytic finding that agreeableness is positively related to life satisfaction (corrected *r* = 0.19). However, the latter is comparable to the unadjusted bivariate association observed in this study (*r* = 0.17); the negative relationship emerged only after controlling for other measured traits and demographics, suggesting the possibility of omitted suppressors in the Steel et al. analysis. Likewise, disadvantageous associations only emerged for openness to experience in our sample when we controlled for other measured traits and demographics. Specifically, when controlling cognitive ability and other covariates, adults who were more open to experience earned and saved less money, experienced more negative emotion, and were less satisfied with their lives.

Why might conscientiousness contribute both to getting ahead in life and to feeling happy about where one is? What seems to tie facets of conscientiousness together is the tendency to act in accordance with long-term, global goals, and standards when there is a temptation to do otherwise (Eisenberg, Duckworth, Spinrad, and Valiente, manuscript submitted; Roberts et al., 2009). In the workplace, conscientious individuals who work hard, complete tasks thoroughly, stay organized, act responsibly, and make decisions carefully are more productive than less conscientious co-workers (Barrick et al., 2001). The same behavioral tendencies may help conscientious individuals maintain healthy social relationships, a key predictor of subjective well-being (Diener and Biswas-Diener, 2008). Conscientious individuals are more likely to avoid unnecessary interpersonal conflict and amend rifts when they do appear (Roberts et al., 2009). These behaviors may explain why conscientiousness predicts how many friends children have better than intelligence or any other Big Five personality trait (Jensen-Campbell and Malcolm, 2007; Allred and Duckworth, manuscript in preparation). In addition, physical health may mediate the association between conscientiousness and success outcomes. Conscientiousness predicts a wide range of physical health outcomes and health promoting behaviors (Deary et al., 2010). More conscientious adults who stay healthier may miss fewer days of work and have lower medical bills, which may benefit both income and wealth. Likewise, healthier individuals tend to be happier (Diener and Biswas-Diener, 2008). Education is another plausible mechanism. More conscientious individuals perform better in school (Poropat, 2009), and it seems likely that academic success leads to better paying jobs and, at least for some individuals, greater subjective well-being.

### Limitations and future directions

There were several limitations of the current investigation. First, income and wealth are incomplete measures of objective success. Nevertheless, earnings and wealth are, as Vaillant and Vaillant (1981) put it, “splendidly objective and easily quantified” (p. 1433). Moreover, while non-pecuniary reasons factor into career and lifestyle choices (Lopez Zadicoff and Lopez Zadicoff, 2005), it is also true that income is positively associated with competing (but, as noted above, less consensually evaluable) metrics of objective success, including occupational prestige (Judge et al., 1999) and educational attainment (Jencks, 1979). Thus, while not the only metric by which to gauge objective success in life, earnings and wealth are in our view better than any other.

A second limitation was the fact that annual Social Security tax contributions, used to assess income in this investigation, are capped at a maximum of approximately $100,000 and therefore do not include the “long right tail” of the income distribution. Indeed, we did not log transform lifetime earnings in our analyses because the untransformed distribution better approximated normality. To address whether the present findings would replicate with more complete measures of income, future studies should make use of alternative objective data sources, such as IRS tax records.

Third, the personality scales in the HRS were relatively brief (i.e., an average of five adjectival descriptors per Big Five trait). While time is always at a premium in large panel surveys, facet-level measures of Big Five personality would permit a more nuanced understanding of how specific behavioral tendencies relate to important life outcomes (De Young et al., 2007). Relatedly, longer measures would increase content validity. For instance, a more extensive conscientiousness measure than was used in the HRS might have included items specifically tapping self-control (Eisenberg et al., manuscript submitted; Roberts et al., 2009).

Finally and most seriously, the cross-sectional design of the current investigation precludes strong causal inferences. It is possible, for example, that objective and subjective success make people more conscientious rather than the other way around. Or, even more likely, there may be reciprocal causality, engendering a virtuous cycle by which conscientiousness leads to objective and subjective success, which further reinforces the same behavioral tendencies. Against the possibility that the causal arrow runs exclusively from success to personality, however, is the fact that personality reaches terminal levels of rank-order stability (exceeding *r* = 0.7) by late adulthood (Roberts and DelVecchio, 2000). Moreover, our findings corroborate prospective, longitudinal studies identifying conscientiousness as a predictor of earnings in smaller convenience samples (Judge et al., 1999; Roberts et al., 2011).

## Conclusion

The current investigation suggests the potential benefit of interventions that increase Big Five conscientiousness in adults. However, interventions administered later in life may not be as cost-effective as those targeting much younger individuals (Heckman, 2006). Moreover, less conscientiousness individuals may not adhere to requirements of an intervention which itself requires conscientious behavior (e.g., Hill and Roberts, 2011). An alternative approach would be to anticipate and accommodate, rather than remediate, domain-specific deficits in conscientious behavior. Recently, Thaler and Sunstein (2008) recommended strategic structuring of the environments in which individuals make important decisions. For instance, making higher contribution rates for retirement savings plans the default option for employees has been shown to improve their savings outcomes (Beshears et al., 2009). Nevertheless, the pervasive benefits of conscientiousness warrant further exploration of interventions aimed at increasing trait-level conscientiousness in adults.

## Conflict of Interest Statement

The authors declare that the research was conducted in the absence of any commercial or financial relationships that could be construed as a potential conflict of interest.

## Footnotes

^1^CFI values over 0.90 indicate a good fit; however, Kenny and McCoach (2003) note that “the CFI tend[s] to demonstrate worse fit as the number of variables in the model increases…Therefore, it appears that the CFI…do[es] not function well with correctly specified models that include a large number of variables” (p. 349). Kenny and McCoach’s “large” model had 40 variables; our models had 40–74 variables.

^2^0.13 * 738,000 = 95,940.

^3^Exp(12.52 + 0.16*1.52 + 0.16*1.52) – exp(12.52) = 171,496.

## References

[B1] Barrick M. R. Mount M. K. Judge T. A. (2001). Personality and performance at the beginning of the new millennium: what do we know and where do we go next? Int. J. Select. Assess. 9, 9–30 10.1111/1468-2389.00160

[B2] Beshears J. Choi J. Laibson D. Madrian B. (2009). The Importance of Default Options for Retirement Savings Outcomes: Evidence from the United States. Available at: http://www.nber.org/chapters/c4539

[B3] Binet A. Simon T. (1916). The Development of Intelligence in Children (The Binet-Simon Scale). Baltimore, MD: Williams & Wilkins Co

[B4] Brandt J. Spencer M. Folstein M. (1988). The telephone interview for cognitive status. Neuropsychiatry Neuropsychol. Behav. Neurol. 1, 111–117

[B5] De Young C. G. Quilty L. C. Peterson J. B. (2007). Between facets and domains: 10 aspects of the Big Five. J. Pers. Soc. Psychol. 93, 880–896 10.1037/0022-3514.93.5.880 17983306

[B6] Deary I. J. Batty G. D. Pattie A. Gale C. R. (2008). More intelligent, more dependable children live longer. Psychol. Sci. 19, 874–880 10.1111/j.1467-9280.2008.02171.x 18947352

[B7] Deary I. J. Weiss A. Batty G. D. (2010). Intelligence and personality as predictors of illness and death: how researchers in differential psychology and chronic disease epidemiology are collaborating to understand and address health inequalities. Psychol. Sci. Public Interest 11, 53–79 10.1177/1529100610387081 26168413

[B8] Diener E. (1984). Subjective well-being. Psychol. Bull. 95, 542–575 10.1037/0033-2909.95.3.542 6399758

[B9] Diener E. Biswas-Diener R. (2008). Happiness: Unlocking the Mysteries of Psychological Wealth. Malden: Blackwell Publishing

[B10] Diener E. Emmons R. A. Larsen R. J. Griffin S. (1985). The satisfaction with life scale. J. Pers. Assess. 49, 71–75 10.1207/s15327752jpa4901_13 16367493

[B11] Farkas G. (2003). Cognitive skills and noncognitive traits and behaviors in stratification processes. Annu. Rev. Sociol. 29, 541–562 10.1146/annurev.soc.29.010202.100023

[B12] Forgeard M. J. C. Jayawickreme E. Kern M. L. Seligman M. E. P. (2011). Doing the right thing: measuring well-being for public policy. Int. J. Wellbeing 1, 79–106

[B13] Gottfredson L. S. (2002). Where and why g matters: not a mystery. Hum. Perform. 15, 25–46 10.1207/S15327043HUP1501&02_03

[B14] Haider S. Solon G. (2000). “Nonrandom Selection in the HRS Social Security Earnings Sample,” Working Paper No. 00-01, RAND Labor and Population Program.

[B15] Heckman J. J. (2006). Skill formation and the economics of investing in disadvantaged children. Science 312, 1900–1902 10.1126/science.1128898 16809525

[B16] Heckman J. J. Stixrud J. Urzua S. (2006). The effects of cognitive and noncognitive abilities on labor market outcomes and social behavior. J. Labor Econ. 24, 411–481 10.1086/504278

[B17] Hemingway E. (1986). The Garden of Eden. New York: Charles Scribner’s Sons

[B18] Herzog A. R. Wallace R. B. (1997). Measures of cognitive functioning in the AHEAD study. J. Gerontol. B Psychol. Sci. Soc. Sci. 52B, 37–48 10.1093/geronb/52B.Special_Issue.37 9215356

[B19] Hill P. L. Roberts B. W. (2011). The role of adherence in the relationship between conscientiousness and perceived health. Health Psychol. 30, 797–804 10.1037/a0023859 21604876 PMC3196269

[B20] Hofstee W. K. B. (2001). “Intelligence and personality: do they mix?,” in Intelligence and Personality: Bridging the Gap in Theory and Measurement, eds Collis J. M. Messick S. (Mahwah: Lawrence Erlbaum Associates Publishers), 43–60

[B21] Jencks C. (1979). Who Gets Ahead? The Determinants of Economic Success in America. New York: Basic Books

[B22] Jensen-Campbell L. A. Malcolm K. T. (2007). The importance of conscientiousness in adolescent interpersonal relationships. Pers. Soc. Psychol. Bull. 33, 368–383 10.1177/0146167206296104 17312318

[B23] John O. P. Naumann L. P. Soto C. J. (2008). “Paradigm shift to the integrative Big Five trait taxonomy: history, measurement, and conceptual issues,” in Handbook of Personality: Theory and Research, eds John O. P. Robins R. W. Pervin L. A. (New York, NY: Guilford Press), 114–158

[B24] John O. P. Srivastava S. (1999). “The Big Five trait taxonomy: history, measurement, and theoretical perspectives,” in Handbook of Personality: Theory and Research, 2nd Edn, eds Pervin L. A. John O. P. (New York, NY: Guilford Press), 102–138

[B25] Judge T. A. Higgins C. A. Thoresen C. J. Barrick M. R. (1999). The Big Five personality traits, general mental ability, and career success across the life span. Pers. Psychol. 52, 621–652 10.1111/j.1744-6570.1999.tb00174.x

[B26] Judge T. A. Ilies R. Dimotakis N. (2010). Are health and happiness the product of wisdom? The relationship of general mental ability to educational and occupational attainment, health, and well-being. J. Appl. Psychol. 95, 454–468 10.1037/a0019084 20476826

[B27] Kahneman D. Deaton A. (2010). High income improves evaluation of life but not emotional well-being. Proc. Natl. Acad. Sci. U.S.A. 107, 16489–16493 10.1073/pnas.1011492107 20823223 PMC2944762

[B28] Kenny D. A. McCoach D. B. (2003). Effect of the number of variables on measures of fit in structural equation modeling. Structural Equation Modeling 10, 333–351 10.1207/S15328007SEM1003_1

[B29] Kern M. L. Friedman H. S. (2008). Do conscientious individuals live longer? A quantitative review. Health Psychol. 27, 505–512 10.1037/0278-6133.27.5.505 18823176

[B30] Kern M. L. Friedman H. S. (2011). Personality and pathways of influence on physical health. Soc. Personal. Psychol. Compass 5, 76–87 10.1111/j.1751-9004.2010.00331.x

[B31] Lachman M. E. Bertrand R. M. (2001). “Personality and the self in midlife,” in Handbook of Midlife Development, ed. Lachman M. E. (New York, NY: John Wiley & Sons), 279–309

[B32] Lopez Zadicoff D. E. Lopez Zadicoff P. D. (2005). The joy of working. Paper Presented at the Asociación Argentina de Economía Política, La Plata

[B33] McCrae R. R. Costa P. T. Jr. (1997). “Conceptions and correlates of openness to experience,” in Handbook of Personality Psychology, eds Hogan R. Johnson J. A. Briggs S. R. (San Diego, CA: Academic Press), 825–847

[B34] Mroczek D. K. Kolarz C. M. (1998). The effect of age on positive and negative affect: a developmental perspective on happiness. J. Pers. Soc. Psychol. 75, 1333–1349 10.1037/0022-3514.75.5.1333 9866191

[B35] Neisser U. Boodoo G. Bouchard T. J. Jr. Boykin A. W. Brody N. Ceci S. J. Urbina S. (1996). Intelligence: knowns and unknowns. Am. Psychol. 51, 77–101 10.1037/0003-066X.51.2.77

[B36] Pavot W. Diener E. (2008). The satisfaction with life scale and the emerging construct of life satisfaction. J. Posit. Psychol. 3, 137–152 10.1080/17439760701756946

[B37] Pavot W. Diener E. (2011). “Personality and happiness: predicting the experience of subjective well-being,” in The Wiley-Blackwell Handbook of Individual Differences, eds Chamorro-Premuzic T. von Stumm S. Furnham A. (Chichester: Wiley-Blackwell), 699–717

[B38] Poropat A. E. (2009). A meta-analysis of the five-factor model of personality and academic performance. Psychol. Bull. 135, 322–338 10.1037/a0014996 19254083

[B39] Roberts B. Jackson J. Duckworth A. L. Von Culin K. (2011). Personality measurement and assessment in large panel surveys. Forum Health Econ. Policy 14, 1–32 10.2202/1558-9544.1268 PMC3595542 23503719

[B40] Roberts B. W. DelVecchio W. F. (2000). The rank-order consistency of personality traits from childhood to old age: a quantitative review of longitudinal studies. Psychol. Bull. 126, 3–25 10.1037/0033-2909.126.1.3 10668348

[B41] Roberts B. W. Jackson J. J. Fayard J. V. Edmonds G. Meints J. (2009). “Conscientiousness,” in Handbook of Individual Differences in Social Behavior, eds Leary M. Hoyle R. (New York, NY: Guilford), 369–381

[B42] Roberts B. W. Kuncel N. R. Shiner R. Caspi A. Goldberg L. R. (2007). The power of personality: the comparative validity of personality traits, socioeconomic status, and cognitive ability for predicting important life outcomes. Pers. Psychol. Sci. 2, 313–345 10.1111/j.1745-6916.2007.00047.x PMC4499872 26151971

[B43] Roberts B. W. Walton K. E. Bogg T. (2005). Conscientiousness and health across the life course. Rev. Gen. Psychol. 9, 156–168 10.1037/1089-2680.9.2.156

[B44] Sacks D. W. Stevenson B. Wolfers J. (2010). Subjective Well-Being, Income, Economic Development and Growth NBER Working Paper series. Cambridge, MA: National Bureau of Economic Research

[B45] Sigelman L. (1981). Is ignorance bliss? A reconsideration of the folk wisdom. Hum. Relat. 34, 965–974 10.1177/001872678103401104

[B46] Smith J. P. (1995). Racial and ethnic differences in wealth in the Health and Retirement Study. J. Hum. Resour. 30(Suppl.), S158–S183 10.2307/146282

[B47] Steel P. Schmidt J. Shultz J. (2008). Refining the relationship between personality and subjective well-being. Psychol. Bull. 134, 138–161 10.1037/0033-2909.134.1.138 18193998

[B48] Thaler R. H. Sunstein C. R. (2008). Nudge. New Haven, CT: Caravan

[B49] Vaillant G. E. Vaillant C. O. (1981). Natural history of male psychological health, X: Work as a predictor of positive mental health. Am. J. Psychiatry 138, 1433–1440 7294211 10.1176/ajp.138.11.1433

[B50] Wechsler D. (1940). Non intellective factors in general intelligence. Psychol. Bull. 37, 444–445

